# Comparative Efficacy and Tolerability of Neoadjuvant Immunotherapy Regimens for Patients with HER2-Positive Breast Cancer: A Network Meta-Analysis

**DOI:** 10.1155/2019/3406972

**Published:** 2019-03-19

**Authors:** Di Wu, Tiejun Chen, Han Jiang, Chongyang Duan, Xinjian Zhang, Yiguang Lin, Size Chen, Fenfang Wu

**Affiliations:** ^1^Department of Central Laboratory, Shenzhen Hospital, Beijing University of Chinese Medicine, Shenzhen, China; ^2^Department of Oncology, The First Affiliated Hospital of Guangdong Pharmaceutical University, Guangzhou, China; ^3^Department of Surgery, The Affiliated Cancer Hospital & Institute of Guangzhou Medical University, Guangzhou, China; ^4^Department of General Surgery, The First Affiliated Hospital of Xiamen University, Xiamen, China; ^5^Department of Biostatistics, Southern Medical University, Guangzhou, China; ^6^Department of Surgery, The Third Affiliated Hospital of Guangzhou University of Chinese Medicine, Guangzhou, China; ^7^School of Life Sciences, University of Technology Sydney, Sydney, NSW, Australia

## Abstract

This network meta-analysis addresses the need for evidence-based best-practice treatment regimens for HER2-positive breast cancer. We compared the relative efficacy and tolerability of currently available HER2-positive neoadjuvant immunotherapy regimens based on systematic searches of available randomized controlled trials (RCTs) data. Based on intention-to-treat principle, pathological complete response (pCR), overall serious adverse events (SAEs), and breast-conserving surgery (BCS) rate were analyzed using random-effect, Bayesian network meta-analysis, and standard pairwise meta-analysis. 16 RCTs (3868 patients) were included. Analyzed treatment regimens were as follows: chemotherapy+trastuzumab+pertuzumab (CTP), trastuzumab emtansine+pertuzumab (MP), chemotherapy+trastuzumab (CT), chemotherapy+pertuzumab (CP), trastuzumab+pertuzumab (TP), chemotherapy+trastuzumab+lapatinib (CTL), and chemotherapy+lapatinib (CL), and chemotherapy (C) alone. We found that, for the chance of achieving pCR, CTP was ranked first (SUCRA: 97%), followed by CTL, MP, and CT (SUCRA: 80%, 75%, and 55%, resp.). MP provided the safest regimen (SUCRA: 97%), then TP, C, and TPC (SUCRA: 82%, 76%, and 47%, resp.). CTL proved the most toxic therapy (SUCRA: 7%). No significant difference between neoadjuvant regimens was identified for BCS. Hormone receptor status did not impact ORs for pCR in any regimen. In conclusion, our findings support CTP as the optimum neoadjuvant regimen for HER2-positive breast cancer, with the best pCR and acceptable toxicity compared with CT. MP provides a therapeutic option for patients with poor performance status.

## 1. Introduction

Worldwide, breast cancer is one of the most common malignancies and the leading cause of death in females, with an estimated 1.7 million new diagnoses annually [1]. Among them, the overexpression of human epidermal growth factor receptor 2 (HER2, also called ErbB2) occurs in roughly 15-20% of breast cancers and is associated with aggressive proliferation and poor prognosis [2]. Until the past decade, increased understanding of the molecular events of HER2-positive oncogenesis has led to the development of a series of HER2-targeted drugs, which have revolutionized the standard of care for HER2-positive disease [3]. To date, four HER2-targeted agents, monoclonal antibody trastuzumab, small-molecule inhibitor lapatinib, anti-HER2 heterodimerization domain antibody pertuzumab, and antibody-drug conjugate trastuzumab emtansine, have been approved for use in patients with metastatic HER2-positive breast cancer, and trials have been conducted, or ongoing, in both adjuvant and neoadjuvant settings.

Neoadjuvant systemic therapy (i.e., regimens commenced before surgery) was once reserved for local advanced breast cancer with the aim of downstaging and achieving operability [4], but it has been routinely delivered in primary operable (early) tumors [5, 6]. Importantly, the individual patient's response to neoadjuvant regimen, designated as pathological complete response (pCR) in the breast and axillary nodes at the time of surgery, is strongly correlated with improved overall survival (OS) and disease-free survival (DFS), particularly in triple-negative and HER2-positive diseases [7]. For this reason, the neoadjuvant approach using pCR as a surrogate endpoint has been adopted to accelerate the approval of new agents for high-risk early-stage breast cancers by the U.S. Food and Drug Administration (FDA) [8, 9] and European Medicines Agency (EMA) [10, 11]. Data from random controlled trials (RCT) has shown that regimens in neoadjuvant settings have similar OS and DFS compared with that in adjuvant trials, and more breast-conserving surgery (BCS) can be performed after neoadjuvant regimens because of tumor shrinkage, thus providing additional support for this approach [12, 13].

The current recommendation regarding neoadjuvant therapy options for HER2-positive breast cancer in National Comprehensive Cancer Network (NCCN) guidelines contains many regimens, including combinational therapies: chemotherapy+trastuzumab+ pertuzumab (CTP), trastuzumab+emtansine+pertuzumab (MP), chemotherapy+ trastuzumab (CT), chemotherapy+pertuzumab (CP), trastuzumab+ pertuzumab (TP), chemotherapy+ trastuzumab+lapatinib (CTL), and chemotherapy plus lapatinib (CL) [14]. With the increasing number of new HER2-directed agents and combination regimens, there is an unmet need to define the optimum neoadjuvant regimens for HER2-positive breast cancer patients. The network meta-analysis enables indirect comparison by using a common comparator when a head-to-head comparison has not been made and combines direct and indirect comparisons to simultaneously compare different regimens with the preservation of randomization in individual trials [15]. Such a technique can improve the precision of the estimate (compared with direct evidence alone) and facilitate the quantification of the relative efficacy of regimens, even if no studies directly compare them [16, 17]. Although it is important to define optimal regimens for HER2-positive breast cancer patients using network meta-analysis, by far, only one network meta-analysis study has been published on the identification of the optimal regimen in patients with early-stage HER2 breast cancer in neoadjuvant setting (data was only updated until August 2012) [18]. A few other nonnetwork meta-analysis studies specifically compared two kinds of neoadjuvant agents using conventional pairwise comparisons [19–23]. Thus, an updated network meta-analysis study is undoubtedly needed.

In this study, we aimed to provide an updated and comprehensive view on the optimum neoadjuvant regimens for patients with HER2-positive breast cancer, through a random-effect network meta-analysis of all relevant randomized evidence comparing the relative efficacy and tolerability of the commonly used neoadjuvant regimens including CTP, MP, CT, CP, TP, CTL, CL, and chemotherapy alone.

## 2. Materials and Methods

### 2.1. Literature Search and Study Selection

Combining the search algorithms* Randomi∗*;* Breast cancer*;* Neoadjuvant*;* HER2/ERBB2,* a systematic search was conducted of articles published until April 2018 from MEDLINE, the Cochrane database, and EMBASE, with no language restriction (see full search terms in eTable 1 in Supplementary Materials). We regarded publications as eligible for inclusion if they were full manuscripts or abstracts of randomized trials that compared the benefits of two or more neoadjuvant regimens for HER2-positive breast cancer. We excluded retrospective or prospective observational cohort trials. Bibliographies of key articles in the field were hand-searched and reviewed for additional candidates. If multiple publications covered the same trial cases, only the most updated or most inclusive publication was included. Our meta-analysis adhered to the Preferred Reporting Items for Systematic Reviews and Meta-Analyses (PRISMA) statement [24].

### 2.2. Outcome Measure and Data Extraction

Our primary outcomes of interest included (1) pCR, defined as the FDA's Guidance for Industry [25], number of patients with no invasive cancer in breast and lymph nodes following completion of neoadjuvant therapy, and regimen-related serious adverse events (SAEs), defined as greater than or equal to grade 3 toxic effects according to the National Cancer Institute Common Terminology Criteria (NCICTC). We only assessed SAEs because grade 1-2 toxicity had lesser clinical significance and was not consistently reported. Secondary outcome was breast-conserving surgery rate (BCS).

Two investigators (W.D. and C.D.) separately selected trials and abstracted data with a prespecified information sheet. Extracted data included characteristics of the trials (acronym of the trial, inclusion period, publication year, country, trial design, randomization process, and stratification), characteristics of the patients (number of patients randomized, disease stage, median age, hormone receptor status, and node positivity), characteristics of the regimens (sequence, dosage, and duration), and outcomes (definition and number of patients using intention-to-treat principle whenever available).

Transitivity (i.e., the assumption that one can validly compare indirectly treatments A and B via one or more anchor treatments) is the fundamental premise underlying network meta-analysis [26, 27]. We examined whether the trials were sufficiently homogenous by comparing population baseline characteristics across the included trials [28].

### 2.3. Quality Assessment

Risk of bias of individual trials was separately assessed by the same investigators using the Cochrane Collaboration's risk-of-bias tool outlined in chapter 8 of the* Cochrane Handbook for Systematic Reviews of Interventions, Version 5.1.0 *[29]. Data and bias discrepancies were resolved by joint discussion to reach consensus.

### 2.4. Data Synthesis and Analysis

We initially performed standard pairwise meta-analyses to assess the available direct relative effects of the neoadjuvant regimens using STATA software version 14.0 (StataCorp, College Station, TX, USA). A random-effects model, which provides more conservative estimated effects, was applied [30]. Because all of the outcomes of interest were dichotomous variables, we calculated the summary effect sizes as odds ratios (OR) with 95% credible intervals (CrI). In these analyses, we used the *I*^2^ index to assess the statistical heterogeneity, with values over 50% indicating significant heterogeneity [31].

To incorporate indirect with direct comparisons, we performed random-effects Bayesian network meta-analyses using Markov chain Monte Carlo methods in WinBUGS software version 1.4.3 (MRC Biostatistics Unit, Cambridge, UK) [16, 32]. This technique combined direct and indirect evidence of all relative treatment effects, provided estimates with maximum power, and allowed the ranking of the various neoadjuvant regimens based on the surface under the cumulative ranking (SUCRA) and the mean ranks [33, 34]. Analyses yielded 50,000 iterations with a burn-in number of 10,000 iterations and a thin interval of 50 to obtain the posterior distributions of the model parameters. Multiple chains (e.g., multiple initial values) were evaluated for each analysis. Convergence of iterations was evaluated by Gelman-Rubin-Brooks statistic [35]. To assess whether there was inconsistency between direct and indirect comparisons, we compared the pooled ORs from the network meta-analysis with corresponding ORs from standard pairwise meta-analysis [36]. Rank probabilities were calculated from proportions of Markov chain cycles. SUCRA for each regimen was calculated from a cumulative ranking probability that a regimen is above a certain ranking [37]. Statistical tests were two-sided and used a significance threshold of* p* < 0.05.

### 2.5. Small-Study Effects and Additional Analyses

We investigated the presence of small-study effects for each outcome by comparison-adjusted funnel plots; comparisons have been directed according to the effectiveness of neoadjuvant regimens, assuming that the more effective regimens are favored in small trials [38, 39]. Potential asymmetry would indicate a form of small-study effects depending on the defined direction, whereas symmetry in the funnel plot would indicate a lack of evidence of small-study effects.

Multiple sensitivity analyses were performed to assess the robustness of the findings. These were based on (1) exclusion of trials using different outcome definitions; (2) exclusion of trials using distinct types of chemotherapy drugs in neoadjuvant therapy; (3) exclusion of trials that did not administered chemotherapy concomitantly with HER2-targeted agents in neoadjuvant therapy; (4) exclusion of trials with high risk of bias in any domain assessed by the Cochrane risk of bias tool; and (5) exclusion of trials published as meeting abstracts.

Additionally, we performed network meta-regression analysis adjusting for the percentage of hormone receptor–positive patients to assess whether the effects of neoadjuvant regimens on pCR were affected by hormone receptor status.

## 3. Results

### 3.1. Study Selection

**Figure 1:**
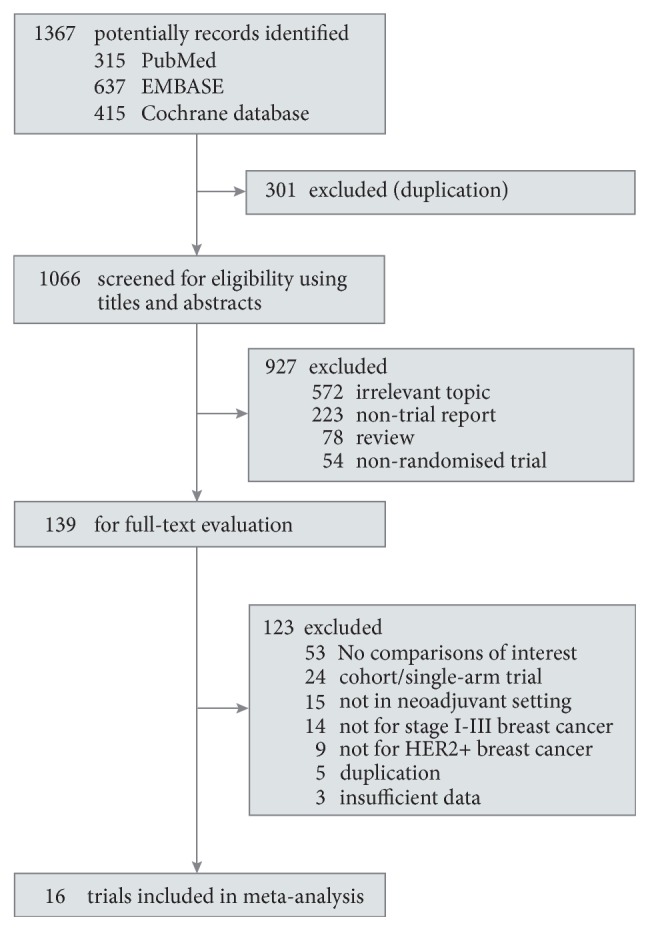
Summary of trial selection for network meta-analysis. HER2 indicates human epidermal growth factor receptor 2.

Of the 1367 potential records that were initially identified by search strategy (Figure 1 and eTable 1 in the Supplementary Materials), 927 were discarded by eligibility screening of titles and abstracts. After further full-text evaluation for the remaining 139 records, 22 publications [40–61] pertaining to 16 distinct neoadjuvant trials were considered eligible for this meta-analysis, which comprised a total of 3868 patients (median number of patients per trial is 240; range: 29-615).

### 3.2. Baseline and Evaluation of Clinical Assumptions

The characteristics of the included trials and patients were presented in eTable 2 in Supplementary Materials. Of the 16 distinct trials, 13 were published as full manuscripts, and the other 3 [46, 47, 55, 59, 60] were in abstract form (of which data was supplemented by records presented on http://ClinicalTrials.gov). These trials mainly took place in North America and Europe and were published or presented between 2005 and 2016. Most trials (12/16) recruited only women, 2 trials [59, 61] included both sexes, and the other 2 [40, 45] did not have a clear description of criteria about sex. This bias was unlikely to influence the results since the majority of participants were women. Eligible patients typically had previously untreated resectable, locally advanced, or inflammatory HER2-positive breast cancer (stage I-IIIC) with adequate baseline function of major organs. The proportion of hormone receptor-positive tumors ranged from 25% to 68% among trials.

The details of the treatment regimen and schedule are presented in eTable 3 in Supplementary Materials. Totally, these 16 trials covered 8 types of neoadjuvant regimens. All trials except two [49, 56] used HER2-targeted agents concomitantly with chemotherapy. In NeoALTTO [49], HER2-targeted agent alone was given for the first six weeks before combination therapy; in ABCGS-24 [56], chemotherapy was used alone for the first eighteen weeks. Over two-thirds of trials (11/16) used polychemotherapy that consisted of anthracycline plus taxane or carboplatin-docetaxel combination, while the others [45, 49, 52, 59, 61] used taxane monochemotherapy in neoadjuvant therapy.

Overall, we found no evidence of important discrepancies regarding trial design, population characteristics, and treatment schedules across the available direct comparisons. Therefore, the assumption of transitivity is likely to hold in the overall data-analysis.

### 3.3. Bias Assessment

Overall risk of bias was low in the included trials (eTable 4 in Supplementary Materials). Most trials (13/16) appropriately reported the method of random sequence generation, whereas in 2 trials [43, 48] there was high risk of bias in terms of allocation concealment. Due to the open-label design of all 16 trials, performance bias might exist [62]. We judged the adequacy of blinding by whether an outcome assessor was masked to treatment assignment, because it was critical to prevent detection bias in assessment of outcomes such as pCR. Nine of the 16 trials assessed the patients' response by a pathologist who was unaware to the treatment, while the other 7 [40, 43, 45, 46, 54, 55, 59] did not present a clear description. None of these trials had evidence of a definite high risk of bias in terms of attrition bias or reporting bias. Additionally, another source of bias was identified: three trials [40, 58, 61] were halted prematurely because of an apparent benefit of a treatment, and 2 [55, 58] had imbalanced baseline characteristics.

### 3.4. Meta-Analysis for Primary Outcomes

#### 3.4.1. Pathological Complete Response

**Figure 2:**
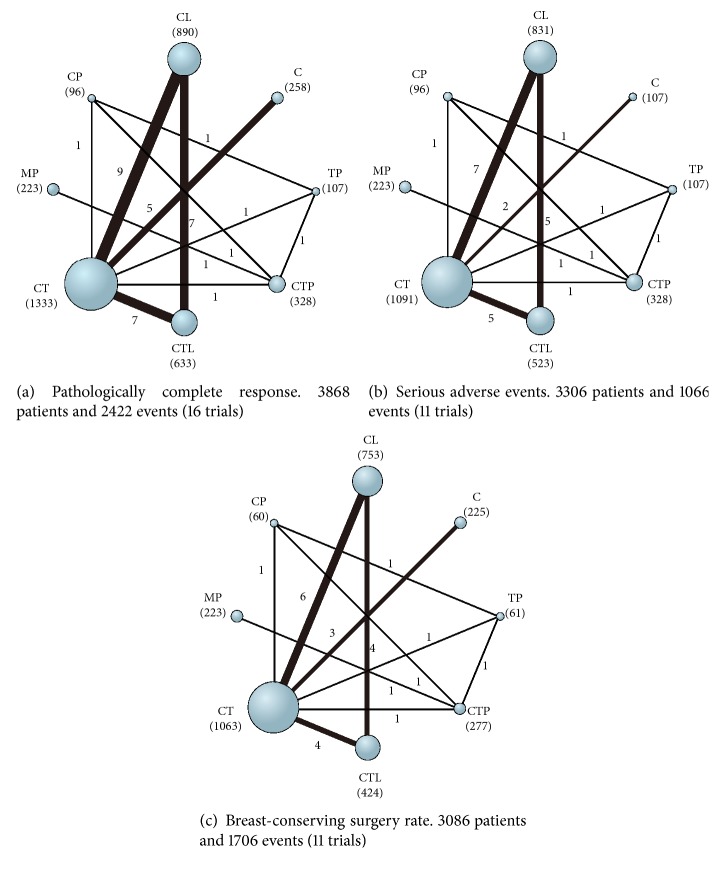
Network diagrams of available treatment comparisons for each outcome. The size of the nodes is proportional to the number of patients (in parentheses) randomized to each treatment, and the width of the lines is proportional to the number of trials (beside the line) comparing the connected treatments. C indicates chemotherapy; CL, chemotherapy plus lapatinib; CP, chemotherapy plus pertuzumab; CT, chemotherapy plus trastuzumab; CTL, chemotherapy plus trastuzumab plus lapatinib; CTP, chemotherapy plus trastuzumab plus pertuzumab; MP, trastuzumab emtansine plus pertuzumab; TP, trastuzumab plus pertuzumab.

**Figure 3:**
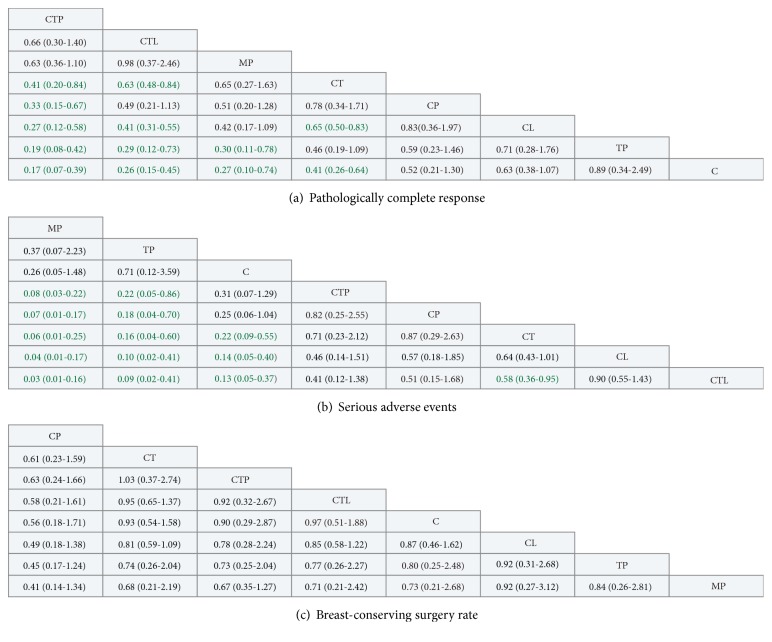
Pooled estimates for all possible treatment effects for each outcome (treatments were ordered by ranking). Effect estimates reflect comparison of the treatment in the row heading being compared to the column heading. Effect estimates of all outcomes are expressed as odds ratios (ORs) with 95% credible intervals. ORs with Bayesian* p* value less than 0.05 are in green. C indicates chemotherapy; CL, chemotherapy plus lapatinib; CP, chemotherapy plus pertuzumab; CT, chemotherapy plus trastuzumab; CTL, chemotherapy plus trastuzumab plus lapatinib; CTP, chemotherapy plus trastuzumab plus pertuzumab; MP, trastuzumab emtansine plus pertuzumab; TP, trastuzumab plus pertuzumab.

All sixteen trials reported data on pCR (3868 patients and 2422 events) and therefore were included in the analysis (Figure 2(a)). All trials except one [45] used pCR definition that there is no invasive cancer in both breast and lymph nodes at the time of surgery. The H2269s trial defined pCR as the absence of invasive cancer in breast only. Of the 28 comparisons included in network meta-analysis, 12 statistically significant differences were identified (Figure 3(a)). CTP was ranked first for the chance of achieving pCR (SUCRA: 97%), with nonsignificant different ORs of 0.66 and 0.63 compared with CTL and MP, and significant differences for the remaining regimens, with ORs ranging from 0.17 to 0.41 (key comparisons include CTP vs CT: OR, 0.41; 95% CrI, 0.20-0.84 and CTL vs CT: OR, 0.63; 95% CrI, 0.48-0.84) (Figure 3(a) and Figure S1A in Supplementary Materials).

Sensitivity analysis with the removal of H2269s did not show any major change in terms of regimen effects or rankings (eTable 5 in Supplementary Materials). Meta-regression analysis on pCR adjusted for the percentage of hormone receptor–positive patients in each trial showed that ORs were not differed by the adjustment (Figure S2 in Supplementary Materials).

#### 3.4.2. Serious Adverse Events

Data on neoadjuvant regimens-related overall SAEs were available in eleven trials (3306 patients and 1066 events) [42, 46, 48, 50–52, 54, 56, 57, 59, 61] (Figure 2(b)). One trial [42] did not report the number of patients with overall SAEs; the trial-specific OR was thus calculated with the sum of the individual serious toxic reactions. Network comparisons showed that MP was ranked as the safest regimen (SUCRA: 97%), with significant differences compared with all regimens except TP and chemotherapy alone (key comparisons include MP vs CTP: OR, 0.08; 95% CrI, 0.03-0.22 and MP vs CT: OR, 0.06; 95% CrI, 0.01-0.25) (Figure 2(b) and Figure S1B in Supplementary Materials). The regimen of CTP ranked fourth (SUCRA: 47%) for SAEs, with no significant differences compared with CT (OR, 1.14; 95% CrI, 0.47-4.26) or chemotherapy alone (OR, 3.20; 95% CrI, 0.78-13.35). There was also no significant difference between CTP and CTL (OR, 0.41; 95% CrI, 0.12-1.38). CTL was more likely to cause SAEs compared with all other regimens (SUCRA: 7%), with five significant differences being identified.

#### 3.4.3. Ranking of Available Regimens

**Figure 4:**
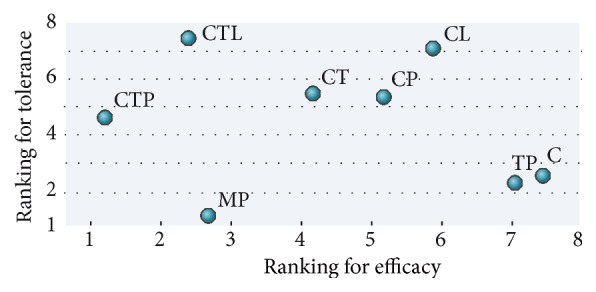
Ranking for pathological complete response and serious adverse events. C indicates chemotherapy; CL, chemotherapy plus lapatinib; CP, chemotherapy plus pertuzumab; CT, chemotherapy plus trastuzumab; CTL, chemotherapy plus trastuzumab plus lapatinib; CTP, chemotherapy plus trastuzumab plus pertuzumab; MP, trastuzumab emtansine plus pertuzumab; TP, trastuzumab plus pertuzumab.

All the eight neoadjuvant regimens were ranked in Figure 4 according to both pCR value and overall safety profile (SAEs). CTP and MP lying in the lower left corner suggested being more favorable for the benefit and toxicity ratio with higher probability of being optimal treatments.

### 3.5. Meta-Analysis for Secondary Outcome

*Breast-Conserving Surgery*. Data from eleven trials (3086 patients and 1706 events) [42, 43, 48, 49, 51, 52, 54, 56–59] were included in the analysis of BCS (Figure 2(c)). The NeoSphere trial [52] only reported the number of patients who transformed to BCS candidates after neoadjuvant treatments, rather than the sum of the patients who underwent BCS. We thus calculated the trial-specific OR with the number of patients who were previously not candidates for BSC and the number of transformed ones for an evaluation of BCS conversion. Network comparisons showed that CP (SUCRA: 90%), CT (SUCRA: 63%), and CTP (SUCRA: 61%) were ranked as the top three regimens with the highest chance of BCS (Figure 2(c) and Figure S1C in Supplementary Materials). However, the findings should be interpreted with caution because all comparisons between the various treatments did not reach statistical significance.

Sensitivity analysis with the removal of NeoSphere trial did not change the rankings of BCS outcome (eTable 6 in Supplementary Materials).

### 3.6. Heterogeneity and Inconsistency

Comparison of results from pairwise meta-analysis and network meta-analysis is presented in eTable 7 in Supplementary Materials, the CIs of all ORs from network comparisons generally included CIs of corresponding ORs from pairwise comparisons, and the point estimates of ORs between the two meta-analyses were similar for each outcome, supporting that there was no important inconsistency between direct and indirect comparisons.

We found no evidence of significant difference between-trial heterogeneity in all comparisons, with the exception of CT versus CL for SAEs analysis (*I*^2^ = 60 %) (eTable 7 in Supplementary Materials).

### 3.7. Small-Study Effects and Additional Analyses

**Figure 5:**
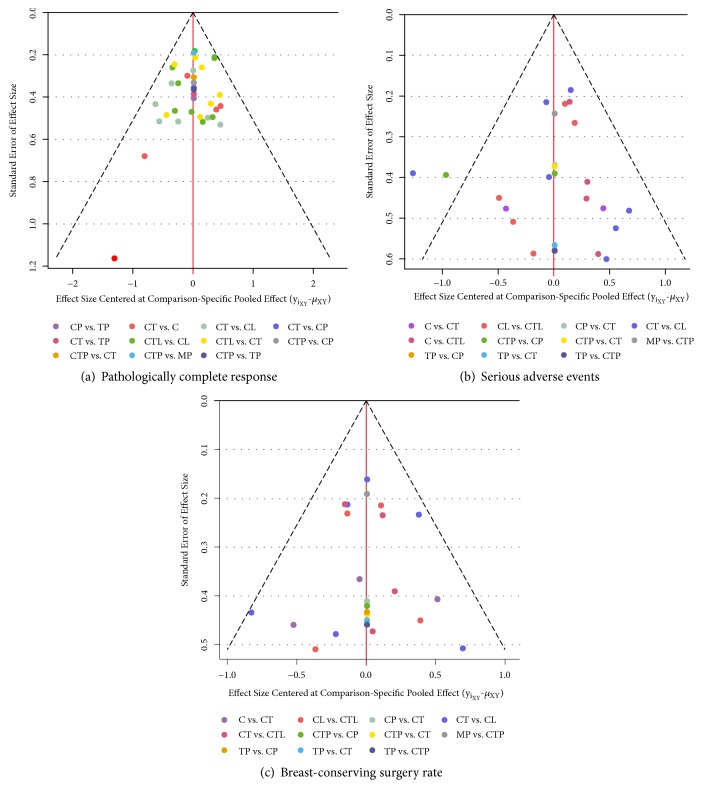
The comparison-adjusted funnel plot for each outcome. The funnel plot is a scatterplot of the treatment effect size vs its standard error. A funnel plot that is asymmetrical with respect to the line of the summary effect (vertical red line) implies that there are differences between the estimates derived from small and large studies. The studies are ordered from best to worst according to treatment effects. Missing (small) studies lying on the right side of the zero line suggest that small studies tend to exaggerate the effectiveness of higher-ranked treatments compared with lower-ranked treatments. Red line represents the null hypothesis that the study-specific effect sizes do not differ from the respective comparison-specific pooled effect estimates.

As shown in Figure 5, the comparison-adjusted funnel plots appeared symmetrical for BCS outcome, but asymmetrical in primary outcomes, largely attributable to the spot located in the lower left corner for pCR that contributed by the H2269s trial [45] and the two outlying spots for SAEs contributed by the NeoALTTO trial [49], suggesting that these trials tended to favor active regimens over comparison-specific weighted average effect [39].

To assess the robustness of our findings, we performed additional sensitivity analyses based on exclusion of trials that did not use HER2-targeted drugs concomitantly with chemotherapy; exclusion of trials that used taxane monochemotherapy only; exclusion of trials that were considered high risk of bias in any bias domain; and exclusion of trials that presented as abstracts. These analyses did not affect the results of primary outcomes (eTables 8–10 in Supplementary Materials).

## 4. Discussion

The present network meta-analysis of 16 randomized controlled trials of 3,868 patients with breast cancer defined optimal neoadjuvant regimens for HER2-positive breast cancer patients by comparison of the relative efficacy and safety profiles of 8 commonly used neoadjuvant regimens, i.e., CTP, MP, CT, CP, TP, CTL, CL, and chemotherapy alone. To the best of our knowledge, this is the most comprehensive and updated study summarizing current randomized evidence on neoadjuvant regimens for HER2-positive breast cancer.

Our findings from this study highlighted the important updates on optimal neoadjuvant regimens for HER2-positive breast cancer patients. Firstly, our data suggested that the dual-HER2 blockade regimen of CTP is currently the most effective neoadjuvant regimen for the chance of achieving pCR, with little additional toxicity compared with CT or chemotherapy alone. This supports the use of neoadjuvant CTP as the first choice for patients with early-stage HER2-positive breast cancer to maximally translate into recurrence-free survival gains. In agreement with our finding, a recent conference report by Nakashoji et al. supported the notion that CTP has the highest probability of achieving pCR (SUCRA = 0.95) [63]. Secondly, although MP ranked only third in achieving pCR, they have the most favorable toxicity profile compared with other treatments and hence might be a suitable regimen option for patients unlikely to tolerate systemic taxane-based chemotherapy. Thirdly, our meta-regression analysis, which considered the potential effect of hormone receptor status on pCR, showed that the findings above were similar after adjustment for hormone receptor status. Finally, these findings are consistent and likely to be robust by assessment of multiple sensitivity analyses considering several patient-, treatment-, and trial-related factors.

More neoadjuvant trials included CTL as an experimental arm (7/16 in our meta-analysis) based on the results of several important preclinical studies [64–66] and adjuvant trials [67, 68] in HER2-postive population. However, most of these neoadjuvant trials (6/7) reported the increased number of grade 3-4 adverse events such as diarrhea, neutropenia, and hepatic toxicity when treated with CTL when compared with CT arm, leading the discontinuation rates to range from 15.3% to 54.5% of CTL arm even after dose adjustment [46, 49, 51, 54, 55, 61]. In our meta-analysis, CTL was ranked as the most toxic neoadjuvant regimen, with significant difference compared with CT. Indeed, a significant increase in the risk of overall SAEs was identified in CTL compared with CTP in an additional analysis using fixed-effect model (Figure S3), despite not being found in our random-effect analysis. The excess benefit (of the chance of achieving pCR) over risk (of experiencing serious adverse events) of CTL might be limited and more favorable in patients with high-risk breast cancer.

We noted no significant differences between neoadjuvant regimens with respect to breast conservation rate. None of the included trials, except for one [69], identified any significant differences for BCS. This trial showed that CTP improved BCS than MP. Nevertheless, our meta-analysis might be underpowered to provide definitive conclusions for ranking of regimen for BCS.

Our study extends findings from primary randomized controlled trials and previous pairwise meta-analyses by systematically synthesizing the entire body of relative and absolute efficacy and safety data. Our findings are partly in keeping with a previous network meta-analysis, reaching a similar conclusion that CTP was the most effective treatment [18]. However, there are several important differences between our study and the network meta-analysis by Nagayama and colleagues. Firstly, our study updated 6 recent randomized trials (LPT 109096, NSABP B41, TRIO-US B07, EORTC 10054, GALGB 40601, and KRISTINE) that were not included in the previous meta-analysis, increasing the sample size by more than a half (3868 versus 2247 patients), and thus providing greater statistical power and more precise estimates. Secondly, our study integrated evidence of a more recent treatment combination-MP into the analysis and, to our knowledge, for the first time represented the network-comparative evidence. Thirdly, rather than using per-protocol (PP) analysis in the previous meta-analysis, where patients who deviated from the protocol are excluded, our analyses were based on the ‘intention-to-treat' principle (ITT). This means that all patients assigned to a group are taken into account, including those who deviated from the protocol for any reasons, for all outcomes when available. In conjunction with randomization, ITT approach is the best to guarantee that the groups of patients being compared have similar characteristics and usually best reflects the effects of treatment because it avoids the dilution due to noncompliance [70, 71]. Therefore, the findings from our study should be considered more conservative [71].

There are certain limitations in our study that merit further discussion. Firstly, same as in the previous study by Nagayama and colleagues, we did not perform meta-analysis on long-term outcomes such as OS and DFS/EFS, because the data accumulation for such outcomes was insufficient. As shown in Table 1, data on long-term outcomes were not available in most of the included trials. Secondly, the number of studies and the number of patients included (totaling 16 trials of 3868 patients) are relatively small. In addition, as shown in Table 1 and Figure 2, 6 out of 16 included studies (38%) included small sample arm/arms that had less than 100 participants (MD Anderson, H2269s, LPT 109096, CHER-LOB, TRIO-US B07, ABCSG-24, and EORTC 10054). As a result, the effect size estimated from those studies might be overestimated owing to lower methodological quality of small studies and possible publication bias [72–74]. Finally, our meta-analysis was based on summary statistics from published randomized trials rather than individual patient data. There might be some covariates at the individual patient level that might affect the treatment outcomes but were not reported. For example, our meta-regression analysis adjusting for hormone receptor status at the study level showed that the ORs on pCR were not different from those without the adjustment. However, such finding might potentially be subject to the ecological fallacy because individual trials did not report ORs comparing patients with and without hormone receptor positivity. Access to and examination of data from individual patients could resolve the problem of missing information on certain prognostic factors and increase the power of the meta-analysis.

## 5. Conclusions

Our findings support that CTP is the currently optimal neoadjuvant immunotherapy regimen for HER2-positive breast cancer, due to the best chance of achieving pCR and relatively modest toxicity profile compared with other treatments. MP has the best tolerability and acceptable efficacy, which may be a therapeutic option for patients with poor performance status. CTL appears to be more toxic than other regimens, whose excess pCR benefits over toxicity were thus more likely achieved in patients with high-risk breast cancer. CP, CL, TP, and chemotherapy alone might not be considered as neoadjuvant therapeutic alternatives.

## Figures and Tables

**Table 1:** Summary of Characteristics and Limitations of all Included Randomized Controlled Trials.

Study	Type	Design	Country	Clinical Stage	Cases (n)	Neoadjuvant treatment	Age	Arm	HER2+ %	HR+ %	Limitations of the Study	Ref
MD Anderson, 2005 & 2007	Peer reviewed	Open-label	United States	II-IIIA	42	CT C	52 48	23 19	100 100	56 58	Small sample size, unoptimal imaging modalities or cancer markers used, unclear description about building of outcome assessment, premature termination	[40, 41]

Pierga, 2010	Peer reviewed	Multicentre, open-label, phase II	France	II-III	120	CT C	47 47	62 58	100 100	55 63	Absence of long-term outcome	[42]

NOAH, 2010 & 2014	Peer reviewed	Multicentre, open-label, phase III	Europe and North America 6 counties	T3N1 or T4 or any T N2-3	235	CT C	NR	117 118	100	35 35	Unclear description about building of outcome assessment.	[43, 44]

H2269s, 2010	Peer reviewed	Open-label	United States	T2-4	29	CT C	50	15 14	100	NR	Small sample size, absence of HR status data, unclear description about building of outcome assessment, different pCR definition used, absence of long-term outcome	[45, 46]

LPT 109096 2011	Abstract	Multicentre, open-label, phase II	United States	T2-4, N0-2	100	CTL CT CL	49 51 51	33 33 34	100	NR	Full-text unavailable, small sample size.	[47]

GeparQuinto–GBG44 2012	Peer reviewed	Multicentre, open-label, phase III	Germany	T1 pNSLN+, T2cN+, T3-4,	615	CT CL	50 50	309 311	100 100	55 56	Unmasked allocation concealment, absence of long-term outcome	[48]

NeoALTTO, 2012 & 2014	Peer reviewed	Multicentre, open-label, phase III	International 25 countries	T2-4	455	CTL CT CL	50 49 50	152 149 154	100 100 100	51 50 52	More patients had to stop treatment due to side-effects in the lapatinib-containing groups	[49, 50]

CHER-LOB, 2012	Peer reviewed	Multicentre, open-label phase IIb	Italy	II-IIIA	121	CTL CT CL	49 50 49	46 36 39	100 100 100	61 58 62	Small sample size, absence of HR status data, absence of long-term outcome	[51]

NeoSphere, 2012 & 2016	Peer reviewed	Multicentre, open-label, phase II	International 19 countries	T2-4	417	CTL TP CT CP	50 49 50 49	107 107 107 96	100 100 100 100	47 47 47 48	Not been identified	[52, 53]

NSABP B41, 2013	Peer reviewed	Muticentre open-label, phase III	North America 3 countries	T2-T3, N0-N2a	519	CTP CT CL	NR	174 181 174	100 100 100	62 67 58	Unmasked allocation concealment, unclear description about building of outcome assessment,	[54]

TRIO-US B07, 2013	Abstract	Multicentre, open-label, phase II	United States	I-III	106	CTL CT CL	NR	58 34 36	100 100 100	NR	Unbalanced baseline characteristic, small sample size, absence of HR status data, absence of long-term outcome	[55]

ABCSG-24, 2013	Peer reviewed	Multicentre open-label, phase III	Austria	T1-4	93	CT C	50 48	44 49	100 100	41 38	Small sample size, key regimen is not used internationally, absence of long-term outcome	[56]

GEICAM, 2014	Peer reviewed	Multicentre, open-label, phase II	Spain	I-III or inflammatory	99	CT CL	49 48	50 52	100 100	60 56	Absence of long-term outcome, small sample size	[57]

EORTC 10054, 2014	Peer reviewed	Multicentre, open-label, phase IIb	Europe 5 countries	IIA-IIC	122	CTL CT CL	49 47 50	52 53 23	100 100 100	52 52 68	Unbalanced baseline characteristic, small sample size, premature termination, absence of long-term outcome	[58]

KRISTINE, 2016	Abstract	Multicentre, open-label, phase III	International, 11 countries	II-IIIC	444	MP CTP	NR	223 221	100 100	51 49	Full-text unavailable	[59, 60]

CALGB 40601, 2016	Peer reviewed	Multicentre, open-label, phase III	United States	II-III	295	CTL CT CL	48 50 50	118 120 67	100 100 100	59 59 58	Premature termination, absence of long-term outcome	[61]

*Note*. C indicates chemotherapy alone; CL, chemotherapy plus lapatinib; CP, chemotherapy plus pertuzumab; CT, chemotherapy plus trastuzumab; CTL, chemotherapy plus trastuzumab plus lapatinib; CTP, chemotherapy plus trastuzumab plus pertuzumab; HER2, human epidermal growth factor receptor-2; HR, hormone receptor; MP, trastuzumab emtansine plus pertuzumab; NR, not reported; TP, trastuzumab plus pertuzumab.

## Data Availability

The data [3 supplementary figures and 10 supplementary tables] used to support the findings of this study are included within the supplementary information file submitted.

## Supplementary Materials

Supplementary Materials The submitted compressed file (Suppl.zip) contains the following supplementary figures and tables:* Figure S1*. Treatment Rankings for Each Outcome;* Figure S2*. Meta-regression Analysis with Adjustment for Hormone Receptor Status for Pathological Complete Response;* Figure S3*. Pooled Estimates for Overall Serious Adverse Events Using Fixed-effect Model.* eTable 1*. Literature Search Strategy;* eTable 2*. Characteristics of Included Trials and Patient Populations;* eTable 3*. Neoadjuvant Treatments in Included Trials;* eTable 4*. Bias Assessment of Included Trials;* eTable 5*. Network Meta-analysis for Pathological Complete Response after Excluding H2269s Trial;* eTable 6*. Network Meta-analysis for Breast-conserving Surgery Rate after Excluding NeoSphere Trial;* eTable 7*. Comparative results from traditional pairwise meta-analysis and network meta-analysis;* eTable 8*. Network Meta-analysis for Primary Outcomes after Excluding the Trials That Did Not Used HER2-targeted Agents Concomitantly with Chemotherapy;* eTable 9*. Network Meta-analysis for Primary Outcomes after Excluding the Trials of High Risk of Bias;* eTable 10*. Network Meta-analysis for Primary Outcomes after Excluding the Trials Presented as Abstracts.
