# Supplementary material for: Comparative Efficacy and Tolerability of Neoadjuvant Immunotherapy Regimens for Patients with HER2-Positive Breast Cancer: A Network Meta-Analysis
Source: J Oncol. 2019 Mar 19;2019:3406972. doi: 10.1155/2019/3406972 (PMC6444249; doi:10.1155/2019/3406972)
Supplement: Supplementary Materials — The submitted compressed file (Suppl.zip) contains the following supplementary figures and tables: Figure S1. Treatment Rankings for Each Outcome; Figure S2. Meta-regression Analysis with Adjustment for Hormone Receptor Status for Pathological Complete Response; Figure S3. Pooled Estimates for Overall Serious Adverse Events Using Fixed-effect Model. eTable 1. Literature Search Strategy; eTable 2. Characteristics of Included Trials and Patient Populations; eTable 3. Neoadjuvant Treatments in Included Trials; eTable 4. Bias Assessment of Included Trials; eTable 5. Network Meta-analysis for Pathological Complete Response after Excluding H2269s Trial; eTable 6. Network Meta-analysis for Breast-conserving Surgery Rate after Excluding NeoSphere Trial; eTable 7. Comparative results from traditional pairwise meta-analysis and network meta-analysis; eTable 8. Network Meta-analysis for Primary Outcomes after Excluding the Trials That Did Not Used HER2-targeted Agents Concomitantly with Chemotherapy; eTable 9. Network Meta-analysis for Primary Outcomes after Excluding the Trials of High Risk of Bias; eTable 10. Network Meta-analysis for Primary Outcomes after Excluding the Trials Presented as Abstracts. [file 3406972.f1.zip › 3406972.f1/eTable 1 Literature Search Strategy.docx]

| **eTable 1.** Literature Search Strategy | | |
| --- | --- | --- |
| **PubMed** (315 records) | **EMBASE** (637 records) | **The Cochrane database** (415 records) |
| 1. breast neoplasm [tiab]  2. breast cancer [tiab]  3. breast carcinoma [tiab]  4. breast tumor [tiab]  5. mammary cancer [tiab]  6. OR/1-5  7. neoadjuvant [tiab]  8. HER2 [tiab]  9. ERBB2 [tiab]  10. OR/8-9  11. randomized controlled trial [pt]  12. randomized [tiab]  13. placebo [tiab]  14. OR/11-13  15. 6 AND 10 AND 14  16. limit 15 to animals  17. limit 16 to humans  18. 16 NOT 17  19. 15 NOT 18 | 1. breast neoplasm:ab,ti  2. breast cancer:ab,ti  3. breast carcinoma:ab,ti  4. breast tumor:ab,ti  5. mammary cancer:ab,ti  6. OR/1-5  7. neoadjuvant:ab,ti  8. HER2:ab,ti  9. ERBB2:ab,ti  10. OR/8-9  11. random*:ab,ti  12. blind*:ab,ti  13. placebo:ab,ti  14. OR/11-13  15. 6 AND 7 AND 10 AND 14  16. limit 15 to animals  17. limit 16 to humans  18. 15 NOT 16  19. 14 NOT 17 | 1. breast neoplasm:ti,ab,kw  2. breast cancer:ti,ab,kw  3. breast carcinoma:ti,ab,kw  4. breast tumor:ti,ab,kw  5. mammary cancer:ti,ab,kw  6. OR/1-5  7. neoadjuvant:ti,ab,kw  8. HER2:ti,ab,kw  9. ERBB2:ti,ab,kw  10. OR/8-9  11. 6 AND 7 AND 10 |
