# Supplementary material for: Comparative Efficacy and Tolerability of Neoadjuvant Immunotherapy Regimens for Patients with HER2-Positive Breast Cancer: A Network Meta-Analysis
Source: J Oncol. 2019 Mar 19;2019:3406972. doi: 10.1155/2019/3406972 (PMC6444249; doi:10.1155/2019/3406972)
Supplement: Supplementary Materials — The submitted compressed file (Suppl.zip) contains the following supplementary figures and tables: Figure S1. Treatment Rankings for Each Outcome; Figure S2. Meta-regression Analysis with Adjustment for Hormone Receptor Status for Pathological Complete Response; Figure S3. Pooled Estimates for Overall Serious Adverse Events Using Fixed-effect Model. eTable 1. Literature Search Strategy; eTable 2. Characteristics of Included Trials and Patient Populations; eTable 3. Neoadjuvant Treatments in Included Trials; eTable 4. Bias Assessment of Included Trials; eTable 5. Network Meta-analysis for Pathological Complete Response after Excluding H2269s Trial; eTable 6. Network Meta-analysis for Breast-conserving Surgery Rate after Excluding NeoSphere Trial; eTable 7. Comparative results from traditional pairwise meta-analysis and network meta-analysis; eTable 8. Network Meta-analysis for Primary Outcomes after Excluding the Trials That Did Not Used HER2-targeted Agents Concomitantly with Chemotherapy; eTable 9. Network Meta-analysis for Primary Outcomes after Excluding the Trials of High Risk of Bias; eTable 10. Network Meta-analysis for Primary Outcomes after Excluding the Trials Presented as Abstracts. [file 3406972.f1.zip › 3406972.f1/eTable 10 Network Meta-analysis for Primary Outcomes after Excluding the Trials Presented as Abstracts.docx]

eTable 10. Network Meta-analysis for Primary Outcomes after Excluding the Trials Presented as Abstracts

| A. Pathological Complete Response | | | | | | |
| --- | --- | --- | --- | --- | --- | --- |
| CTP (SUCRA: 97 %) | -- | -- | -- | -- | -- | -- |
| 0.68 (0.29-1.56) | CTL (SUCRA: 85 %) | -- | -- | -- | -- | -- |
| 0.42 (0.19-0.91) | 0.62 (0.45-0.86) | CT (SUCRA: 62 %) | -- | -- | -- | -- |
| 0.30 (0.14-0.73) | 0.48 (0.19-1.22) | 0.78 (0.33-1.83) | CP (SUCRA: 46 %) | -- | -- | -- |
| 0.28 (0.12-0.62) | 0.41 (0.29-0.57) | 0.66 (0.48-0.86) | 0.85 (0.34-2.09) | CL (SUCRA: 35 %) | -- | -- |
| 0.19 (0.08-0.45) | 0.28 (0.11-0.72) | 0.46 (0.19-1.08) | 0.59 (0.23-1.48) | 0.69 (0.27-1.74) | TP (SUCRA: 16 %) | -- |
| 0.18 (0.07-0.41) | 0.26 (0.14-0.45) | 0.41 (0.25-0.66) | 0.53 (0.20-1.38) | 0.62 (0.36-1.10) | 0.91 (0.33-2.46) | C (SUCRA: 9 %) |

| B. Serious Adverse Events | | | | | | |
| --- | --- | --- | --- | --- | --- | --- |
| TP (SUCRA: 93 %) | -- | -- | -- | -- | -- | -- |
| 0.72 (0.11-4.03) | C (SUCRA: 87 %) | -- | -- | -- | -- | -- |
| 0.22 (0.04-0.95) | 0.31 (0.07-1.45) | CTP (SUCRA: 55 %) | -- | -- | -- | -- |
| 0.19 (0.04-0.78) | 0.26 (0.05-1.17) | 0.83 (0.24-2.94) | CP (SUCRA: 46 %) | -- | -- | -- |
| 0.16 (0.03-0.64) | 0.22 (0.08-0.58) | 0.71 (0.21-2.46) | 0.86 (0.26-2.80) | CT (SUCRA: 44 %) | -- | -- |
| 0.10 (0.02-0.44) | 0.14 (0.05-0.40) | 0.44 (0.12-1.68) | 0.54 (0.15-1.94) | 0.62 (0.38-1.03) | CL (SUCRA: 16 %) | -- |
| 0.09 (0.02-0.39) | 0.12 (0.04-0.37) | 0.39 (0.11-1.48) | 0.48 (0.13-1.77) | 0.56 (0.31-0.97) | 0.90 (0.50-1.54) | CTL (SUCRA: 9 %) |

C indicates chemotherapy alone; CL, chemotherapy plus lapatinib; CP, chemotherapy plus pertuzumab; CT, chemotherapy plus trastuzumab; CTL, chemotherapy plus trastuzumab plus lapatinib; CTP, chemotherapy plus trastuzumab plus pertuzumab; TP, trastuzumab plus pertuzumab.
