# Supplementary material for: Comparative Efficacy and Tolerability of Neoadjuvant Immunotherapy Regimens for Patients with HER2-Positive Breast Cancer: A Network Meta-Analysis
Source: J Oncol. 2019 Mar 19;2019:3406972. doi: 10.1155/2019/3406972 (PMC6444249; doi:10.1155/2019/3406972)
Supplement: Supplementary Materials — The submitted compressed file (Suppl.zip) contains the following supplementary figures and tables: Figure S1. Treatment Rankings for Each Outcome; Figure S2. Meta-regression Analysis with Adjustment for Hormone Receptor Status for Pathological Complete Response; Figure S3. Pooled Estimates for Overall Serious Adverse Events Using Fixed-effect Model. eTable 1. Literature Search Strategy; eTable 2. Characteristics of Included Trials and Patient Populations; eTable 3. Neoadjuvant Treatments in Included Trials; eTable 4. Bias Assessment of Included Trials; eTable 5. Network Meta-analysis for Pathological Complete Response after Excluding H2269s Trial; eTable 6. Network Meta-analysis for Breast-conserving Surgery Rate after Excluding NeoSphere Trial; eTable 7. Comparative results from traditional pairwise meta-analysis and network meta-analysis; eTable 8. Network Meta-analysis for Primary Outcomes after Excluding the Trials That Did Not Used HER2-targeted Agents Concomitantly with Chemotherapy; eTable 9. Network Meta-analysis for Primary Outcomes after Excluding the Trials of High Risk of Bias; eTable 10. Network Meta-analysis for Primary Outcomes after Excluding the Trials Presented as Abstracts. [file 3406972.f1.zip › 3406972.f1/eTable 2 Characteristic of Included Trials and Patient Populations.docx]

| **eTable 2.** Characteristics of Included Trials and Patient Populations | | | | | | | | | | | | | | | | | | | | |
| --- | --- | --- | --- | --- | --- | --- | --- | --- | --- | --- | --- | --- | --- | --- | --- | --- | --- | --- | --- | --- |
| No. | Study | Type | Design | Country | Stage | No. | Neoadjuvant  treatment | Age | Arm | HER2+  % | HR+  % | Stage % | | Tumor status % | | Nodal status % | | | | REF |
|  |  |  |  |  |  |  |  |  |  |  |  | II | III | T2 | T3/4 | N0 | N1 | N2 | N3 |  |
| 1 | MD Anderson,  2005 and 2007 | Peer reviewed | Open-label | United States | II-IIIA | 42 | CT  C | 52  48 | 23  19 | 100  100 | 56  58 | --  -- | --  -- | 36  31 | 12  10 | 24  17 | 29  29 | 2  0 | --  -- |  |
| 2 | Pierga,  2010 | Peer reviewed | Multicentre,  open-label,  phase II | France | II-III | 120 | CT  C | 47  47 | 62  58 | 100  100 | 55  63 | --  -- | --  -- | 27  23 | 25  26 | 21  15 | 28  30 | 3  3 | --  -- |  |
| 3 | NOAH,  2010 and 2014 | Peer reviewed | Multicentre,  open-label, phase III | Europe and North America  6 counties | T3N1 or T4 or any T N2-3 | 235 | CT  C | NR | 117  118 | 100 | 35  35 | --  -- | --  -- | --  -- | --  -- | 7  8 | 21  23 | 21  20 | --  -- |  |
| 4 | H2269s,  2010 | Peer reviewed | Open-label | United States | T2-4 | 29 | CT  C | 50 | 15  14 | 100 | NR | --  -- | --  -- | --  -- | --  -- | --  -- | --  -- | --  -- | --  -- |  |
| 5 | LPT 109096, 2011 | Abstract | Multicentre,  open-label,  phase II | United States | T2-4, N0-2 | 100 | CTL  CT  CL | 49  51  51 | 33  33  34 | 100 | NR | --  -- | --  -- | --  -- | --  -- | --  -- | --  -- | --  -- | --  -- |  |
| 6 | GeparQuinto–GBG44,  2012 | Peer reviewed | Multicentre,  open-label,  phase III | Germany | T1 pNSLN+, T2cN+, T3-4, | 615 | CT  CL | 50  50 | 309  311 | 100  100 | 55  56 | --  -- | --  -- | 29  30 | 12  12 | 15  15 | 34  34 | --  -- | --  -- |  |
| 7 | NeoALTTO,  2012 and 2014 | Peer reviewed | Multicentre,  open-label,  phase III | International  25 countries | T2-4 | 455 | CTL  CT  CL | 50  49  50 | 152  149  154 | 100  100  100 | 51  50  52 | --  --  -- | --  --  -- | 21  15  20 | 12  17  13 | 28  28  28 | | 5  5  5 | |  |
| 8 | CHER-LOB,  2012 | Peer reviewed | Multicentre,  open-label phase IIb | Italy | II-IIIA | 121 | CTL  CT  CL | 49  50  49 | 46  36  39 | 100  100  100 | 61  58  62 | 31  25  26 | 7  5  6 | --  --  -- | --  --  -- | --  --  -- | --  --  -- | --  --  -- | --  --  -- |  |
| 9 | NeoSphere,  2012 and 2016 | Peer reviewed | Multicentre,  open-label, phase II | International  19 countries | T2-4 | 417 | CTL  TP  CT  CP | 50  49  50  49 | 107  107  107  96 | 100  100  100  100 | 47  47  47  48 | --  --  --  -- | --  --  --  -- | --  --  --  -- | --  --  --  -- | 7  7  7  7 | 13  11  12  10 | 5  5  5  5 | 0  1  1  1 |  |
| 10 | NSABP B41,  2013 | Peer reviewed | Muticentre  open-label, phase III | North America  3 countries | T2-T3, N0-N2a | 519 | CTP  CT  CL | NR | 174  181  174 | 100  100  100 | 62  67  58 | --  --  -- | --  --  -- | --  --  -- | --  --  -- | --  --  -- | --  --  -- | --  --  -- | --  --  -- |  |
| 11 | TRIO-US B07,  2013 | Abstract | Multicentre,  open-label,  phase II | United States | I-III | 106 | CTL  CT  CL | NR | 58  34  36 | 100  100  100 | NR | --  --  -- | --  --  -- | --  --  -- | --  --  -- | --  --  -- | --  --  -- | --  --  -- | --  --  -- |  |
| 12 | ABCSG-24,  2013 | Peer reviewed | Multicentre  open-label,  phase III | Austria | T1-4 | 93 | CT  C | 50  48 | 44  49 | 100  100 | 41  38 | --  -- | --  -- | 27  26 | 12  17 | --  -- | --  -- | --  -- | --  -- |  |
| 13 | GEICAM,  2014 | Peer reviewed | Multicentre,  open-label,  phase II | Spain | I-III or inflammatory | 99 | CT  CL | 49  48 | 50  52 | 100  100 | 60  56 | --  -- | --  -- | 31  29 | 13  15 | 13  19 | 35  32 | 2  1 | --  -- |  |
| 14 | EORTC 10054,  2014 | Peer reviewed | Multicentre,  open-label,  phase IIb | Europe  5 countries | IIA-IIC | 122 | CTL  CT  CL | 49  47  50 | 52  53  23 | 100  100  100 | 52  52  68 | --  --  -- | --  --  -- | 23  20  9 | 18  24  9 | 16  14  6 | 24  26  11 | 2  2  2 | 1  1  1 |  |
| 15 | KRISTINE,  2016 | Abstract | Multicentre,  open-label, phase III | International,  11 countries | II-IIIC | 444 | MP  CTP | NR | 223  221 | 100  100 | 51  49 | --  -- | --  -- | --  -- | --  -- | --  -- | --  -- | --  -- | --  -- |  |
| 16 | CALGB 40601,  2016 | Peer reviewed | Multicentre,  open-label,  phase III | United States | II-III | 295 | CTL  CT  CL | 48  50  50 | 118  120  67 | 100  100  100 | 59  59  58 | 65  65  37 | 30  30  14 | --  --  -- | --  --  -- | --  --  -- | --  --  -- | --  --  -- | --  --  -- |  |
| C indicates chemotherapy alone; CL, chemotherapy plus lapatinib; CP, chemotherapy plus pertuzumab; CT, chemotherapy plus trastuzumab; CTL, chemotherapy plus trastuzumab plus lapatinib; CTP, chemotherapy plus trastuzumab plus pertuzumab; HER2, human epidermal growth factor receptor-2; HR, hormone receptor; MP, trastuzumab emtansine plus pertuzumab; NR, not available; TP, trastuzumab plus pertuzumab. | | | | | | | | | | | | | | | | | | | | |
