# Supplementary material for: Comparative Efficacy and Tolerability of Neoadjuvant Immunotherapy Regimens for Patients with HER2-Positive Breast Cancer: A Network Meta-Analysis
Source: J Oncol. 2019 Mar 19;2019:3406972. doi: 10.1155/2019/3406972 (PMC6444249; doi:10.1155/2019/3406972)
Supplement: Supplementary Materials — The submitted compressed file (Suppl.zip) contains the following supplementary figures and tables: Figure S1. Treatment Rankings for Each Outcome; Figure S2. Meta-regression Analysis with Adjustment for Hormone Receptor Status for Pathological Complete Response; Figure S3. Pooled Estimates for Overall Serious Adverse Events Using Fixed-effect Model. eTable 1. Literature Search Strategy; eTable 2. Characteristics of Included Trials and Patient Populations; eTable 3. Neoadjuvant Treatments in Included Trials; eTable 4. Bias Assessment of Included Trials; eTable 5. Network Meta-analysis for Pathological Complete Response after Excluding H2269s Trial; eTable 6. Network Meta-analysis for Breast-conserving Surgery Rate after Excluding NeoSphere Trial; eTable 7. Comparative results from traditional pairwise meta-analysis and network meta-analysis; eTable 8. Network Meta-analysis for Primary Outcomes after Excluding the Trials That Did Not Used HER2-targeted Agents Concomitantly with Chemotherapy; eTable 9. Network Meta-analysis for Primary Outcomes after Excluding the Trials of High Risk of Bias; eTable 10. Network Meta-analysis for Primary Outcomes after Excluding the Trials Presented as Abstracts. [file 3406972.f1.zip › 3406972.f1/eTable 3 Neoadjuvant Treatments of Included Trial.docx]

| **eTable 3.** Neoadjuvant Treatments in Included Trials | | | | | | | | | | | | |
| --- | --- | --- | --- | --- | --- | --- | --- | --- | --- | --- | --- | --- |
| No. | Study | Neoadjuvant treatment | | | | | | |  | Adjuvant treatment | | |
|  |  | Chemotherapy | | |  | Anti-HER2 treatment | | |  |  |  |  |
|  |  | Agents | Dose^a^ | Schedule^b^ |  | Agents | Dose | Schedule^b^ |  | Agents | Dose | Schedule^b`^ |
| 1 | MD Anderson,  2005 and 2007 | Paclitaxel →  FEC^c^ | 225 mg/m^2^ →  500, 45, 500 mg/m^2^ | Q3w 4c  Q3w 4c |  | T | 2 mg/kg^d^ | Q1w 24w |  | None | None | None |
| 2 | Pierga,  2010 | EC →  docetaxel | 75, 750 mg/m^2^ →  100 mg/m^2^ | Q3w 4c  Q3w 4c |  | T | 6 mg/kg^e^ | Q3w 4c |  | T for both arms | 6 mg/kg | Q3w 18c |
| 3 | NOAH,  2010 and 2014 | AP→  Paclitaxel →  CMF | 45, 100 mg/m^2^ →  175 mg/m^2^→  600, 40, 600 mg/m^2^ | Q3w 3c  Q3w 3c  Q4w 3c |  | T | 6 mg/kg^e^ | Q3w 10c |  | T for all arms | 6 mg/kg | Q3w 16c |
| 4 | H2269s,  2010 | Docetaxel + carboplatin | 75 + 75 mg/m^2^ | Q3w 4c |  | T | 2 mg/kg^d^ | Q1w 12w |  | T for both arms | 2 mg/kg  2 mg/kg | Q1w 40w  Q1w 52w |
| 5 | LPT 109096,  2011 | FEC→  paclitaxel | 500, 75, 500 mg/m^2^→  80 mg/m^2^ | Q3w 4c  Q1w 12w |  | TL  T  L | 2 mg/kg^d^ + 750 mg^f^  2 mg/kg^d^  1250 mg | Q1w 14w + Q1d 14w  Q1w 14w  Q1d 14w |  | NR | NR | NR |
| 6 | GeparQuinto–GBG44,  2012 | EC →  docetaxel | 90, 600 mg/m^2^ →  100 mg/m^2^ | Q3w 4c  Q3w 4c |  | T  L | 6 mg/kg^e^  1000 mg | Q3w 8c  Q1d 24w |  | T for both arms | 6 mg/kg  1000 mg | Q3w 16c |
| 7 | NeoALTTO,  2012 and 2014 | Paclitaxel | 80 mg/m^2^ | Q1w 12w |  | TL  T  L | 2 mg/kg^d^ + 750 mg^f^  2 mg/kg^d^  1500 mg | Q1w 18w + Q1d 18w  Q1w 18w  Q1d 18w |  | FEC →  T for all arms | 500, 100, 500 mg/m^2^ →  2 mg/kg | Q3w 3c  Q1w 34w |
| 8 | CHER-LOB,  2012 | Paclitaxel →  FEC | 80 mg/m^2^ →  600, 75, 600 mg/m^2^ | Q1w 12w  Q3w 4c |  | TL  T  L | 2 mg/kg^d^ + 1000 mg  2 mg/kg^d^  1500 mg | Q1w 26w + Q1d 26w  Q1w 26w  Q1d 26w |  | T | As neoadjuvant therapy |  |
| 9 | NeoSphere,  2012 and 2016 | Docetaxel | 75-100 mg/m^2^  (escalated to 100 if tolerated) | Q3w 4c |  | TP  T  P | 6 mg/kg^e^ + 420 mg^g^  6 mg/kg^e^  420 mg^g^ | Q3w 4c + Q3w 4c  Q3w 4c  Q3w 4c |  | FEC + T for arm a  b and d  docetaxel + T for arm c | 500, 100, 500 mg/m^2^ + 6 mg/kg  75 mg/m^2^ + 6 mg/kg | Q3w c3 + Q3w 16c  Q3w c4 + Q3w 16c |
| 10 | NSABP B41,  2013 | doxorubicin-cyclophosphamide →  paclitaxel | 60, 600 mg/m^2^ →  80 mg/m^2^ | Q3w 4c  Q4w 4c |  | TL  T  T | 2 mg/kg^d^ + 1000 mg  2 mg/kg^d^  1500 mg | Q1w 12w  Q1w 12w  Q1w 12w |  | T for all arms | 6 mg/kg | Q3w 16c |
| 11 | TRIO-US B07,  2013 | doxorubicin-cyclophosphamide → docetaxel | 75, 600 mg/m^2^ | Q3w 6c |  | TL  T  L | 6 mg/kg^e^ + 1000 mg  6 mg/kg^e^  1000 mg | Q3w 9c + Q3w 9c  Q3w 9c  Q3w 9c |  | NR | NR | NR |
| 12 | ABCSG-24,  2013 | ED ± capecitabine | 75, 75 mg/m^2^  ± 1000 mg/m^2^ | Q3w 6c |  | T | 6 mg/kg^e^ | Q3w 6c |  | NR | NR | NR |
| 13 | GEICAM,  2014 | EC →  docetaxel | 90, 600 mg/m^2^ →  100 mg/m^2^ | Q3w 4c  Q3w 4c |  | T  L | 6 mg/kg^e^  1250 mg | Q3w 4c  Q1d 12w |  | NR | NR | NR |
| 14 | EORTC 10054,  2014 | Docetaxel →  FEC | 100 mg/m^2^→  500, 100, 500 mg/m^2^ | Q3w 3c  Q3w 3c |  | TL  T  L | 2 mg/kg^d^ + 1000 mg  2 mg/kg^d^  1000 mg | Q1w 9w + Q1d 9w  Q1w 9w  Q1d 9w |  | NR | NR | NR |
| 15 | KRISTINE,  2016 | Docetaxel + carboplatin | 75 + 75 mg/m^2^ | Q3w 6c |  | MP  TP | 3.6 mg/kg + 420 mg^g^  6 mg/kg^e^ + 420 mg^g^ | Q3w 6c + Q3w 6c  Q3w 6c |  | NR | NR | NR |
| 16 | CALGB 40601,  2016 | Paclitaxel | 80 mg/m^2^ | Q1w 16w |  | TL  T  L | 2 mg/kg^d^ + 1000 mg  2 mg/kg^d^  1500 mg | Q1w 16w + Q1d 16w  Q1w 16w  Q1d 16w |  | Doxorubicin +  cyclophosphamide →  T for all arms | 60 + 600 mg/m^2^  →  2 mg/kg | Q3w 4c  Q1w 36w |
| AP indicates doxorubicin plus paclitaxel; CMF, cyclophosphamide plus methotrexate plus fluorouracil; EC, epirubicin plus cyclophosphamide; ED, epirubicin plus docetaxel; FEC, fluorouracil plus epirubicin plus cyclophosphamide; L, lapatinib; MP, trastuzumab emtansine plus pertuzumab; NR, not reported; P, pertuzumab; T, trastuzumab; TL, trastuzumab plus lapatinib; TP, trastuzumab plus pertuzumab.  ^a^ Day of administration is day 1 unless otherwise indicated.  ^b^ c, cycle; d, day; Q, every; w, week.  ^c^ Fluorouracil in this study was used in day 1 and 4.  ^d^ Trastuzumab was given with a loading dose of 4 mg/kg.  ^e^ Trastuzumab was given with a loading dose of 8 mg/kg.  ^f^ Lapatinib was given with a loading dose of 1000 mg.  ^g^ Pertuzumab was given with a loading dose of 840 mg. | | | | | | | | | | | | |
