# Supplementary material for: Comparative Efficacy and Tolerability of Neoadjuvant Immunotherapy Regimens for Patients with HER2-Positive Breast Cancer: A Network Meta-Analysis
Source: J Oncol. 2019 Mar 19;2019:3406972. doi: 10.1155/2019/3406972 (PMC6444249; doi:10.1155/2019/3406972)
Supplement: Supplementary Materials — The submitted compressed file (Suppl.zip) contains the following supplementary figures and tables: Figure S1. Treatment Rankings for Each Outcome; Figure S2. Meta-regression Analysis with Adjustment for Hormone Receptor Status for Pathological Complete Response; Figure S3. Pooled Estimates for Overall Serious Adverse Events Using Fixed-effect Model. eTable 1. Literature Search Strategy; eTable 2. Characteristics of Included Trials and Patient Populations; eTable 3. Neoadjuvant Treatments in Included Trials; eTable 4. Bias Assessment of Included Trials; eTable 5. Network Meta-analysis for Pathological Complete Response after Excluding H2269s Trial; eTable 6. Network Meta-analysis for Breast-conserving Surgery Rate after Excluding NeoSphere Trial; eTable 7. Comparative results from traditional pairwise meta-analysis and network meta-analysis; eTable 8. Network Meta-analysis for Primary Outcomes after Excluding the Trials That Did Not Used HER2-targeted Agents Concomitantly with Chemotherapy; eTable 9. Network Meta-analysis for Primary Outcomes after Excluding the Trials of High Risk of Bias; eTable 10. Network Meta-analysis for Primary Outcomes after Excluding the Trials Presented as Abstracts. [file 3406972.f1.zip › 3406972.f1/eTable 4 Bias Assessment of Included Trials.docx]

| **eTable 4.** Bias Assessment of Included Trials | | | | | | |
| --- | --- | --- | --- | --- | --- | --- |
| Study | Sequence generation  (Selection bias) | Allocation concealment  (Selection bias) | Blinding of outcome assessment  (Detection bias) | Incomplete data addressed  (Attrition bias) | Selective reporting  (reporting bias) | Other source of bias^e^ |
| MD Anderson,  2005 and 2007 | Low risk  (Central randomization) | Unclear risk^c^  (Not detailed) | Unclear risk^d^  (Not detailed) | Low risk  (Reasons for missing data were sufficient, and numbers were balanced) | Low risk  (Protocol was not available, but  all pre-specified outcomes were included) | Premature termination, due to the superiority of trastuzumab plus chemotherapy |
| Pierga,  2010 | Low risk  (Central randomization) | Low risk  (Central allocation) | Low risk  (Investigators assessing outcome were masked) | Low risk  (Reasons for missing data were sufficient, and numbers were balanced) | Low risk  (Protocol was not available, but  all pre-specified outcomes were included) | Low risk |
| NOAH,  2010 and 2014 | Low risk  (Minimization) | Low risk  (Central allocation) | Unclear risk^d^  (Not detailed) | Low risk  (Reasons for missing data were sufficient, and numbers were balanced) | Low risk  (Protocol was not available, but  all pre-specified outcomes were included) | Low risk |
| H2269s,  2010 | Unclear risk^b^  (Not detailed) | Unclear risk^c^  (Not detailed) | Unclear risk^d^  (Not detailed) | Low risk  (Reasons for missing data were sufficient, and numbers were balanced) | Low risk  (Protocol was not available, but  all pre-specified outcomes were included) | Low risk |
| LPT 109096^a^,  2011 | Low risk  (Computer random number generator ) | Unclear risk^c^  (Not detailed) | Unclear risk^d^  (Not detailed) | Unclear risk  (Not detailed) | Unclear risk  (Not detailed) | Unclear risk |
| GeparQuinto–GBG44,  2012 | Low risk  (Minimization) | High risk  (Neither patients nor investigators were masked to treatment) | Low risk  (Investigators assessing outcome were masked) | Low risk  (Reasons for missing data were sufficient, and numbers were balanced) | Low risk  (Protocol was not available, but  all pre-specified outcomes were included) | Low risk |
| NeoALTTO,  2012 and 2014 | Low risk  (Minimization) | Low risk  (Central allocation) | Low risk  (Investigators assessing outcome were masked) | Low risk  (Reasons for missing data were sufficient, and numbers were balanced) | Low risk  (Protocol was not available, but  all pre-specified outcomes were included) | Low risk |
| CHER-LOB,  2012 | Low risk  (Computer random number generator ) | Low risk  (Central allocation) | Low risk  (Investigators assessing outcome were masked) | Low risk  (Reasons for missing data were sufficient, and numbers were balanced) | Low risk  (Protocol was not available, but  all pre-specified outcomes were included) | Low risk |
| NeoSphere,  2012 and 2016 | Low risk  (Central randomization using an interactive voice response system and dynamic allocation) | Low risk  (Central allocation) | Low risk  (Investigators assessing outcome were masked) | Low risk  (Reasons for missing data were sufficient, and numbers were balanced) | Low risk  (Protocol was available, and  all pre-specified outcomes were included) | Low risk |
| NSABP B41,  2013 | Low risk  (Biased-coin algorithm) | High risk  (Neither patients nor investigators were masked to treatment) | Unclear risk^d^  (Not detailed) | Low risk  (Reasons for missing data were sufficient, and numbers were balanced) | Low risk  (Protocol was not available, but  all pre-specified outcomes were included) | Low risk |
| TRIO-US B07^a^,  2013 | Unclear risk^b^  (Not detailed) | Unclear risk^c^  (Not detailed) | Unclear risk^d^  (Not detailed) | Unclear risk  (Not detailed) | Unclear risk  (Not detailed) | Unbalanced baseline characteristic (Percentage of patients in each arm:  45, 27, 28%) |
| ABCSG-24,  2013 | Low risk  (Computer random number generator) | Low risk  (Central allocation) | Low risk  (Investigators assessing outcome were masked) | Low risk  (Reasons for missing data were sufficient, and numbers were balanced) | Low risk  (Protocol was not available, but  all pre-specified outcomes were included) | Low risk |
| GEICAM,  2014 | Low risk  (Central randomization) | Low risk  (Central allocation) | Low risk  (Investigators assessing outcome were masked) | Low risk  (Reasons for missing data were sufficient, and numbers were balanced) | Low risk  (Protocol was not available, but  all pre-specified outcomes were included) | Low risk |
| EORTC 10054,  2015 | Low risk  (Minimization) | Low risk  (Central allocation) | Low risk  (Investigators assessing outcome were masked) | Low risk  (Reasons for missing data were sufficient, and numbers were balanced) | Low risk  (Protocol was available, and  all pre-specified outcomes were included) | Unbalanced baseline characteristic (Percentage of patients in each arm: 41, 41, 18%)  Premature termination for lapatinib-only arm, due to the Inferiority to other arms. |
| KRISTINE,  2016 | Low risk  (Central randomization using an interactive  web-based response system) | Low risk  (Central allocation) | Unclear risk^d^  (Not detailed) | Low risk  (Reasons for missing data were sufficient, and numbers were balanced) | Low risk  (Protocol was available, and  all pre-specified outcomes were included) | Low risk |
| CALGB 40601,  2016 | Low risk  (Minimization) | Low risk  (Central allocation) | Low risk  (Investigators assessing outcome were masked) | Low risk  (Reasons for missing data were sufficient, and numbers were balanced) | Low risk  (Protocol was not available, but  all pre-specified outcomes were included) | Premature termination for lapatinib-only arm, due to the Inferiority to other arms. |
| ^a^ Data was from abstracts, presentation slides or records presented on ClinicalTrials.gov. Therefore, all bias domains were considered unclear risk of bias.  ^b^ Random sequence was not reported but it was considered unclear risk of bias, not high risk of bias, based on the description of randomization process.  ^c^ Allocation concealment was not reported but the trial method was considered high quality. Therefore, the allocation concealment was judged as unclear risk of bias rather than high risk of bias.  ^d^ Blinding outcome assessment was not reported and detection bias was considered unclear risk of bias.  ^e^ Other source of bias was evaluated according to the Cochrane risk of bias assessment tool (“other potential threats to validity” section). | | | | | | |
