# Supplementary material for: Comparative Efficacy and Tolerability of Neoadjuvant Immunotherapy Regimens for Patients with HER2-Positive Breast Cancer: A Network Meta-Analysis
Source: J Oncol. 2019 Mar 19;2019:3406972. doi: 10.1155/2019/3406972 (PMC6444249; doi:10.1155/2019/3406972)
Supplement: Supplementary Materials — The submitted compressed file (Suppl.zip) contains the following supplementary figures and tables: Figure S1. Treatment Rankings for Each Outcome; Figure S2. Meta-regression Analysis with Adjustment for Hormone Receptor Status for Pathological Complete Response; Figure S3. Pooled Estimates for Overall Serious Adverse Events Using Fixed-effect Model. eTable 1. Literature Search Strategy; eTable 2. Characteristics of Included Trials and Patient Populations; eTable 3. Neoadjuvant Treatments in Included Trials; eTable 4. Bias Assessment of Included Trials; eTable 5. Network Meta-analysis for Pathological Complete Response after Excluding H2269s Trial; eTable 6. Network Meta-analysis for Breast-conserving Surgery Rate after Excluding NeoSphere Trial; eTable 7. Comparative results from traditional pairwise meta-analysis and network meta-analysis; eTable 8. Network Meta-analysis for Primary Outcomes after Excluding the Trials That Did Not Used HER2-targeted Agents Concomitantly with Chemotherapy; eTable 9. Network Meta-analysis for Primary Outcomes after Excluding the Trials of High Risk of Bias; eTable 10. Network Meta-analysis for Primary Outcomes after Excluding the Trials Presented as Abstracts. [file 3406972.f1.zip › 3406972.f1/eTable 5 Network Meta-analysis for Pathologically Complete Response after Excluding H2269s Trial.docx]

eTable 5. Network Meta-analysis for Pathological Complete Response after Excluding H2269s Trial

| CTP (SUCRA: 97 %) | -- | -- | -- | -- | -- | -- | -- |
| --- | --- | --- | --- | --- | --- | --- | --- |
| 0.66 (0.30-1.41) | CTL (SUCRA: 80 %) | -- | -- | -- | -- | -- | -- |
| 0.64 (0.36-1.09) | 0.96 (0.38-2.51) | MP (SUCRA: 75 %) | -- | -- | -- | -- | -- |
| 0.41 (0.20-0.86) | 0.64 (0.49-0.84) | 0.66 (0.27-1.61) | CT (SUCRA: 56 %) | -- | -- | -- | -- |
| 0.33 (0.15-0.69) | 0.49 (0.21-1.17) | 0.51 (0.19-1.29) | 0.78 (0.34-1.71) | CP (SUCRA: 41 %) | -- | -- | -- |
| 0.27 (0.12-0.58) | 0.41 (0.31-0.55) | 0.43 (0.16-1.07) | 0.65 (0.50-0.83) | 0.84 (0.36-1.93) | CL (SUCRA: 30 %) | -- | -- |
| 0.19 (0.08-0.43) | 0.29 (0.11-0.69) | 0.30 (0.11-0.79) | 0.46 (0.19-1.09) | 0.58 (0.24-1.41) | 0.70 (0.28-1.69) | TP (SUCRA: 14 %) | -- |
| 0.17 (0.07-0.41) | 0.26 (0.16-0.45) | 0.27 (0.10-0.75) | 0.41 (0.26-0.64) | 0.54 (0.21-1.34) | 0.64 (0.37-1.08) | 0.91 (0.35-2.47) | C (SUCRA: 8 %) |

C indicates chemotherapy; CL, chemotherapy plus lapatinib; CP, chemotherapy plus pertuzumab; CT, chemotherapy plus trastuzumab; CTL, chemotherapy plus trastuzumab plus lapatinib; CTP, chemotherapy plus trastuzumab plus pertuzumab; MP, trastuzumab emtansine plus pertuzumab; TP, trastuzumab plus pertuzumab.
