# Supplementary material for: Comparative Efficacy and Tolerability of Neoadjuvant Immunotherapy Regimens for Patients with HER2-Positive Breast Cancer: A Network Meta-Analysis
Source: J Oncol. 2019 Mar 19;2019:3406972. doi: 10.1155/2019/3406972 (PMC6444249; doi:10.1155/2019/3406972)
Supplement: Supplementary Materials — The submitted compressed file (Suppl.zip) contains the following supplementary figures and tables: Figure S1. Treatment Rankings for Each Outcome; Figure S2. Meta-regression Analysis with Adjustment for Hormone Receptor Status for Pathological Complete Response; Figure S3. Pooled Estimates for Overall Serious Adverse Events Using Fixed-effect Model. eTable 1. Literature Search Strategy; eTable 2. Characteristics of Included Trials and Patient Populations; eTable 3. Neoadjuvant Treatments in Included Trials; eTable 4. Bias Assessment of Included Trials; eTable 5. Network Meta-analysis for Pathological Complete Response after Excluding H2269s Trial; eTable 6. Network Meta-analysis for Breast-conserving Surgery Rate after Excluding NeoSphere Trial; eTable 7. Comparative results from traditional pairwise meta-analysis and network meta-analysis; eTable 8. Network Meta-analysis for Primary Outcomes after Excluding the Trials That Did Not Used HER2-targeted Agents Concomitantly with Chemotherapy; eTable 9. Network Meta-analysis for Primary Outcomes after Excluding the Trials of High Risk of Bias; eTable 10. Network Meta-analysis for Primary Outcomes after Excluding the Trials Presented as Abstracts. [file 3406972.f1.zip › 3406972.f1/eTable 6 Network Meta-analysis for Breast-conserving Surgery Rate after Excluding NeoSphere Trial.docx]

eTable 6. Network Meta-analysis for Breast-conserving Surgery Rate after Excluding NeoSphere Trial

| CT (SUCRA: 75 %) | -- | -- | -- |
| --- | --- | --- | --- |
| 0.95 (0.66-1.37) | CTL (SUCRA: 58 %) | -- | -- |
| 0.94 (0.55-1.63) | 0.99 (0.52-1.95) | C (SUCRA: 51 %) | -- |
| 0.81 (0.55-1.09) | 0.85 (0.58-1.24) | 0.86 (0.46-1.57) | CL (SUCRA: 16 %) |

C indicates chemotherapy; CL, chemotherapy plus lapatinib; CT, chemotherapy plus trastuzumab; CTL, chemotherapy plus trastuzumab plus lapatinib.
