# Supplementary material for: Comparative Efficacy and Tolerability of Neoadjuvant Immunotherapy Regimens for Patients with HER2-Positive Breast Cancer: A Network Meta-Analysis
Source: J Oncol. 2019 Mar 19;2019:3406972. doi: 10.1155/2019/3406972 (PMC6444249; doi:10.1155/2019/3406972)
Supplement: Supplementary Materials — The submitted compressed file (Suppl.zip) contains the following supplementary figures and tables: Figure S1. Treatment Rankings for Each Outcome; Figure S2. Meta-regression Analysis with Adjustment for Hormone Receptor Status for Pathological Complete Response; Figure S3. Pooled Estimates for Overall Serious Adverse Events Using Fixed-effect Model. eTable 1. Literature Search Strategy; eTable 2. Characteristics of Included Trials and Patient Populations; eTable 3. Neoadjuvant Treatments in Included Trials; eTable 4. Bias Assessment of Included Trials; eTable 5. Network Meta-analysis for Pathological Complete Response after Excluding H2269s Trial; eTable 6. Network Meta-analysis for Breast-conserving Surgery Rate after Excluding NeoSphere Trial; eTable 7. Comparative results from traditional pairwise meta-analysis and network meta-analysis; eTable 8. Network Meta-analysis for Primary Outcomes after Excluding the Trials That Did Not Used HER2-targeted Agents Concomitantly with Chemotherapy; eTable 9. Network Meta-analysis for Primary Outcomes after Excluding the Trials of High Risk of Bias; eTable 10. Network Meta-analysis for Primary Outcomes after Excluding the Trials Presented as Abstracts. [file 3406972.f1.zip › 3406972.f1/eTable 7 Comparative results from traditional pairwise meta-analysis and network meta-analysis.docx]

| **eTable 7**. Comparative results from traditional pairwise meta-analysis and network meta-analysis | | | | | | | |
| --- | --- | --- | --- | --- | --- | --- | --- |
| Outcomes | No. of study | No. of events/total | | Pairwise meta-analysis | | | Network meta-analysis |
|  |  | Arm 1 | Arm 2 | *I*^2^ (%) | *P* value | Pooled OR | Pooled OR |
| Pathological complete response | | | | | | |  |
| CTP vs MP | 1 | 98/221 | 124/223 | – | 0.02 | 0.64 (0.44-0.93) | 0.63 (0.36-1.10) |
| CTP vs CT | 1 | 65/107 | 84/107 | – | 0.005 | 0.42 (0.23-0.77) | 0.41 (0.20-0.84) |
| CTP vs CP | 1 | 65/107 | 79/96 | – | 0.001 | 0.33 (0.17 -0.64) | 0.33 (0.15 -0.67) |
| CTP vs TP | 1 | 65/107 | 95/107 | – | < 0.001 | 0.20 (0.10-0.40) | 0.19 (0.08 -0.42) |
| CTL vs CT | 7 | 307/633 | 362/606 | 0 | < 0.001 | 0.63 (0.50-0.79) | 0.63 (0.48-0.84) |
| CTL vs CL | 7 | 307/633 | 359/527 | 0 | < 0.001 | 0.43 (0.33-0.55) | 0.41 (0.31-0.55) |
| CT vs CP | 1 | 84/107 | 79/96 | – | 0.50 | 0.79 (0.39-1.58) | 0.78 (0.34-1.71) |
| CT vs CL | 9 | 605/965 | 640/890 | 4.8 | < 0.001 | 0.67 (0.54-0.82) | 0.65 (0.50-0.83) |
| CT vs TP | 1 | 84/107 | 95/107 | – | 0.045 | 0.46 (0.22-0.98) | 0.46 (0.29-1.09) |
| CT vs C | 5 | 164/261 | 206/258 | 11.7 | < 0.001 | 0.43 (0.28-0.67) | 0.41 (0.26-0.64) |
| CP vs TP | 1 | 79/96 | 95/107 | – | 0.19 | 0.59 (0.27-1.30) | 0.59 (0.23-1.16) |
| Serious adverse event | | | | | | |  |
| CTP vs MP | 1 | 141/221 | 29/223 | – | < 0.001 | 11.79 (7.32-19.00) | 12.08 (4.47-32.79) |
| CTP vs CT | 1 | 15/107 | 20/107 | – | 0.36 | 0.71 (0.34-1.47) | 0.71 (0.23-2.12) |
| CTP vs CP | 1 | 15/107 | 16/96 | – | 0.61 | 0.82 (0.38 -1.75) | 0.82 (0.25-2.55) |
| CTP vs TP | 1 | 15/107 | 4/107 | – | 0.01 | 4.20 (1.35-13.10) | 4.53 (1.16-20.38) |
| CTL vs CT | 5 | 183/523 | 171/519 | 49.2 | 0.02 | 1.76 (1.09-2.82) | 1.73 (1.06-2.79) |
| CTL vs CL | 5 | 183/523 | 173/468 | 0 | 0.89 | 1.02 (0.77-1.36) | 1.11 (0.70-1.83) |
| CT vs CP | 1 | 20/107 | 16/96 | – | 0.71 | 1.15 (0.56-2.37) | 1.14 (0.38-3.39) |
| CT vs CL | 7 | 222/878 | 288/831 | 60.1 | 0.04 | 0.65 (0.43-0.99) | 0.64 (0.43-1.01) |
| CT vs TP | 1 | 20/107 | 4/107 | – | 0.002 | 5.92 (1.95-17.98) | 6.33 (1.68-28.53) |
| CT vs C | 2 | 89/106 | 59/107 | 40.3 | 0.001 | 4.33 (1.84-10.18) | 4.50 (1.82-11.22) |
| CP vs TP | 1 | 16/96 | 4/107 | – | 0.005 | 5.15 (1.66-16.01) | 5.49 (1.42-24.49) |
| Breast-conserving surgery | | | | | | |  |
| CTP vs MP | 1 | 109/221 | 132/223 | – | 0.04 | 0.67 (0.46-0.98) | 0.67 (0.35-1.27) |
| CTP vs CT | 1 | 43/56 | 48/62 | – | 0.94 | 0.97 (0.41-2.28) | 0.97 (0.36-2.71) |
| CTP vs CP | 1 | 43/56 | 41/60 | – | 0.31 | 1.53 (0.67-3.50) | 1.60 (0.60-4.09) |
| CTP vs TP | 1 | 43/56 | 50/61 | – | 0.49 | 0.73 (0.30-1.79) | 0.73 (0.25-2.04) |
| CTL vs CT | 4 | 216/424 | 214/419 | 0 | 0.96 | 1.00 (0.77-1.33) | 1.05 (0.73-1.54) |
| CTL vs CL | 4 | 216/424 | 210/390 | 0 | 0.56 | 0.92 (0.70-1.22) | 0.85 (0.58-1.22) |
| CT vs CP | 1 | 48/62 | 41/60 | – | 0.26 | 1.59 (0.71-3.36) | 1.64 (0.63-4.27) |
| CT vs CL | 6 | 372/778 | 398/753 | 42.6 | 0.17 | 0.81 (0.60-1.10) | 0.81 (0.59-1.09) |
| CT vs TP | 1 | 48/62 | 50/61 | – | 0.53 | 0.75 (0.31-1.83) | 0.74 (0.26-2.04) |
| CT vs C | 3 | 147/223 | 150/225 | 31.0 | 0.83 | 0.94 (0.54-1.64) | 0.93 (0.54-1.58) |
| CP vs TP | 1 | 41/60 | 50/61 | – | 0.09 | 0.48 (0.20-1.11) | 0.45 (0.17-1.24) |
| C indicates chemotherapy alone; CL, chemotherapy plus lapatinib; CP, chemotherapy plus pertuzumab; CT, chemotherapy plus trastuzumab; CTL, chemotherapy plus trastuzumab plus lapatinib; CTP, chemotherapy plus trastuzumab plus pertuzumab; MP, trastuzumab emtansine plus pertuzumab; TP, trastuzumab plus pertuzumab. | | | | | | | |
