# Supplementary material for: Comparative Efficacy and Tolerability of Neoadjuvant Immunotherapy Regimens for Patients with HER2-Positive Breast Cancer: A Network Meta-Analysis
Source: J Oncol. 2019 Mar 19;2019:3406972. doi: 10.1155/2019/3406972 (PMC6444249; doi:10.1155/2019/3406972)
Supplement: Supplementary Materials — The submitted compressed file (Suppl.zip) contains the following supplementary figures and tables: Figure S1. Treatment Rankings for Each Outcome; Figure S2. Meta-regression Analysis with Adjustment for Hormone Receptor Status for Pathological Complete Response; Figure S3. Pooled Estimates for Overall Serious Adverse Events Using Fixed-effect Model. eTable 1. Literature Search Strategy; eTable 2. Characteristics of Included Trials and Patient Populations; eTable 3. Neoadjuvant Treatments in Included Trials; eTable 4. Bias Assessment of Included Trials; eTable 5. Network Meta-analysis for Pathological Complete Response after Excluding H2269s Trial; eTable 6. Network Meta-analysis for Breast-conserving Surgery Rate after Excluding NeoSphere Trial; eTable 7. Comparative results from traditional pairwise meta-analysis and network meta-analysis; eTable 8. Network Meta-analysis for Primary Outcomes after Excluding the Trials That Did Not Used HER2-targeted Agents Concomitantly with Chemotherapy; eTable 9. Network Meta-analysis for Primary Outcomes after Excluding the Trials of High Risk of Bias; eTable 10. Network Meta-analysis for Primary Outcomes after Excluding the Trials Presented as Abstracts. [file 3406972.f1.zip › 3406972.f1/eTable 8 Network Meta-analysis for Primary Outcomes after Excluding the Trials That Did Not Used HER2-targeted Agents Concomitantly with Chemotherapy.docx]

**eTable 8**. Network Meta-analysis for Primary Outcomes after Excluding the Trials That Did Not Used HER2-targeted Agents Concomitantly with Chemotherapy

| A. Pathologically Complete Response | | | | | | | |
| --- | --- | --- | --- | --- | --- | --- | --- |
| CTP (SUCRA: 98 %) | -- | -- | -- | -- | -- | -- | -- |
| 0.60 (0.27-1.33) | CTL (SUCRA: 78 %) | -- | -- | -- | -- | -- | -- |
| 0.63 (0.36-1.11) | 1.04 (0.40-2.88) | MP (SUCRA: 75 %) | -- | -- | -- | -- | -- |
| 0.42 (0.20-0.89) | 0.70 (0.52-0.95) | 0.67 (0.25-1.68) | CT (SUCRA: 56 %) | -- | -- | -- | -- |
| 0.33 (0.15-0.69) | 0.55 (0.22-1.34) | 0.52 (0.20-1.34) | 0.79 (0.34-1.78) | CP (SUCRA: 42 %) | -- | -- | -- |
| 0.27 (0.12-0.61) | 0.45 (0.32-0.62) | 0.43 (0.16-1.14) | 0.65 (0.48-0.85) | 0.83 (0.34-2.04) | CL (SUCRA: 30 %) | -- | -- |
| 0.20 (0.08-0.42) | 0.32 (0.12-0.79) | 0.31 (0.11-0.79) | 0.46 (0.18-1.09) | 0.58 (0.24-1.44) | 0.71 (0.27-1.75) | TP (SUCRA: 15 %) | -- |
| 0.16 (0.06-0.38) | 0.26 (0.14-0.48) | 0.25 (0.09-0.72) | 0.38 (0.22-0.64) | 0.48 (0.18-1.32) | 0.58 (0.32-1.05) | 0.81 (0.30-2.23) | C (SUCRA: 7 %) |

| B. Serious Adverse Events | | | | | | | |
| --- | --- | --- | --- | --- | --- | --- | --- |
| MP (SUCRA: 98 %) | -- | -- | -- | -- | -- | -- | -- |
| 0.37 (0.10-1.63) | TP (SUCRA: 85 %) | -- | -- | -- | -- | -- | -- |
| 0.17 (0.04-0.73) | 0.45 (0.08-2.07) | C (SUCRA: 70 %) | -- | -- | -- | -- | -- |
| 0.08 (0.04-0.16) | 0.22 (0.06-0.72) | 0.48 (0.13-1.94) | CTP (SUCRA: 50 %) | -- | -- | -- | -- |
| 0.07 (0.02-0.20) | 0.18 (0.04-0.59) | 0.40 (0.10-1.53) | 0.82 (0.34-1.94) | CP (SUCRA: 39 %) | -- | -- | -- |
| 0.06 (0.02-0.17) | 0.16 (0.04-0.49) | 0.34 (0.12-1.01) | 0.72 (0.30-1.65) | 0.87 (0.37-2.04) | CT (SUCRA: 36 %) | -- | -- |
| 0.04 (0.01-0.13) | 0.12 (0.03-0.39) | 0.26 (0.09-0.80) | 0.54 (0.22-1.34) | 0.66 (0.27-1.64) | 0.75 (0.55-1.07) | CL (SUCRA: 15 %) | -- |
| 0.04 (0.01-0.12) | 0.11 (0.03-0.36) | 0.23 (0.07-0.72) | 0.48 (0.19-1.22) | 0.59 (0.24-1.48) | 0.68 (0.45-1.01) | 0.90 (0.58-1.33) | CTL (SUCRA: 7 %) |

C indicates chemotherapy alone; CL, chemotherapy plus lapatinib; CP, chemotherapy plus pertuzumab; CT, chemotherapy plus trastuzumab; CTL, chemotherapy plus trastuzumab plus lapatinib; CTP, chemotherapy plus trastuzumab plus pertuzumab; MP, trastuzumab emtansine plus pertuzumab; TP, trastuzumab plus pertuzumab.
