# Supplementary material for: Comparative Efficacy and Tolerability of Neoadjuvant Immunotherapy Regimens for Patients with HER2-Positive Breast Cancer: A Network Meta-Analysis
Source: J Oncol. 2019 Mar 19;2019:3406972. doi: 10.1155/2019/3406972 (PMC6444249; doi:10.1155/2019/3406972)
Supplement: Supplementary Materials — The submitted compressed file (Suppl.zip) contains the following supplementary figures and tables: Figure S1. Treatment Rankings for Each Outcome; Figure S2. Meta-regression Analysis with Adjustment for Hormone Receptor Status for Pathological Complete Response; Figure S3. Pooled Estimates for Overall Serious Adverse Events Using Fixed-effect Model. eTable 1. Literature Search Strategy; eTable 2. Characteristics of Included Trials and Patient Populations; eTable 3. Neoadjuvant Treatments in Included Trials; eTable 4. Bias Assessment of Included Trials; eTable 5. Network Meta-analysis for Pathological Complete Response after Excluding H2269s Trial; eTable 6. Network Meta-analysis for Breast-conserving Surgery Rate after Excluding NeoSphere Trial; eTable 7. Comparative results from traditional pairwise meta-analysis and network meta-analysis; eTable 8. Network Meta-analysis for Primary Outcomes after Excluding the Trials That Did Not Used HER2-targeted Agents Concomitantly with Chemotherapy; eTable 9. Network Meta-analysis for Primary Outcomes after Excluding the Trials of High Risk of Bias; eTable 10. Network Meta-analysis for Primary Outcomes after Excluding the Trials Presented as Abstracts. [file 3406972.f1.zip › 3406972.f1/eTable 9 Network Meta-analysis for Primary Outcomes after Excluding the Trials of High Risk of Bias.docx]

eTable 9. Network Meta-analysis for Primary Outcomes after Excluding the Trials of High Risk of Bias

| A. Pathologically Complete Response | | | | | | | |
| --- | --- | --- | --- | --- | --- | --- | --- |
| CTP (SUCRA: 97 %) | -- | -- | -- | -- | -- | -- | -- |
| 0.66 (0.29-1.47) | CTL (SUCRA: 80 %) | -- | -- | -- | -- | -- | -- |
| 0.63 (0.36-1.11) | 0.96 (0.37-2.56) | MP (SUCRA: 75 %) | -- | -- | -- | -- | -- |
| 0.30 (0.11-0.79) | 0.63 (0.46-0.89) | 0.66 (0.26-1.62) | CT (SUCRA: 55 %) | -- | -- | -- | -- |
| 0.32 (0.15-0.70) | 0.49 (0.20-1.16) | 0.50 (0.20-1.62) | 0.77 (0.34-1.71) | CP (SUCRA: 42 %) | -- | -- | -- |
| 0.23 (0.10-0.72) | 0.35 (0.25-0.50) | 0.36 (0.14-0.96) | 0.55 (0.39-0.77) | 0.72 (0.30-1.70) | CL (SUCRA: 25 %) | -- | -- |
| 0.19 (0.08-0.44) | 0.29 (0.11-0.72) | 0.31 (0.11-0.79) | 0.45 (0.19-1.07) | 0.60 (0.24-1.41) | 0.83 (0.33-2.06) | TP (SUCRA: 15 %) | -- |
| 0.17 (0.07-0.40) | 0.26 (0.15-0.45) | 0.27 (0.10-0.72) | 0.41 (0.26-0.63) | 0.53 (0.21-1.32) | 0.75 (0.42-1.30) | 0.90 (0.35-2.39) | C (SUCRA: 9 %) |

| B. Serious Adverse Events | | | | | | | |
| --- | --- | --- | --- | --- | --- | --- | --- |
| MP (SUCRA: 95 %) | -- | -- | -- | -- | -- | -- | -- |
| 0.37 (0.04-4.32) | TP (SUCRA: 82 %) | -- | -- | -- | -- | -- | -- |
| 0.26 (0.02-2.84) | 0.71 (0.08-5.44) | C (SUCRA: 76 %) | -- | -- | -- | -- | -- |
| 0.22 (0.03-1.23) | 0.22 (0.03-1.23) | 0.32 (0.05-2.11) | CTP (SUCRA: 46 %) | -- | -- | -- | -- |
| 0.07 (0.01-0.59) | 0.18 (0.03-0.99) | 0.26 (0.03-0.47) | 0.81 (0.17-3.86) | CP (SUCRA: 39 %) | -- | -- | -- |
| 0.06 (0.01-0.50) | 0.16 (0.02-0.88) | 0.22 (0.07-0.69) | 0.70 (0.16-3.36) | 0.85 (0.19-4.11) | CT (SUCRA: 37 %) | -- | -- |
| 0.04 (0.01-0.40) | 0.10 (0.01-0.67) | 0.14 (0.04-0.57) | 0.44 (0.09-2.61) | 0.54 (0.10-3.18) | 0.63 (0.32-1.38) | CL (SUCRA: 17 %) | -- |
| 0.03 (0.01-0.31) | 0.08 (0.01-0.55) | 0.12 (0.03-0.47) | 0.37 (0.07-2.15) | 0.45 (0.08-2.60) | 0.53 (0.24-1.18) | 0.84 (0.36-1.74) | CTL (SUCRA: 9 %) |

Abbreviation: C, chemotherapy; CL, chemotherapy plus lapatinib; CP, chemotherapy plus pertuzumab; CT, chemotherapy plus trastuzumab; CTL, chemotherapy plus trastuzumab plus lapatinib; CTP, chemotherapy plus trastuzumab plus pertuzumab; MP, trastuzumab emtansine plus pertuzumab; TP, trastuzumab plus pertuzumab.
